# STAT1 deficiency redirects IFN signalling toward suppression of TLR response through a feedback activation of STAT3

**DOI:** 10.1038/srep13414

**Published:** 2015-08-24

**Authors:** Hun Sik Kim, Dong Chan Kim, Hong-Mi Kim, Hyung-Joon Kwon, Soon Jae Kwon, Suk-Jo Kang, Sun Chang Kim, Go-Eun Choi

**Affiliations:** 1 Department of Biomedical Sciences, University of Ulsan College of Medicine, Seoul 138-736, Korea; 2 Cellular Dysfunction Research Center, University of Ulsan College of Medicine, Seoul 138-736, Korea; 3 Department of Microbiology, University of Ulsan College of Medicine, Seoul 138-736, Korea; 4 Department of Biological Sciences, Korea Advanced Institute of Science and Technology, Daejeon, 305–701, Korea

## Abstract

Interferons (IFNs) potentiate macrophage activation typically via a STAT1-dependent pathway. Recent studies suggest a functioning of STAT1-independent pathway in the regulation of gene expression by IFN-γ, thus pointing to the diversity in cellular responses to IFNs. Many functions of IFNs rely on cross-regulation of the responses to exogenous inflammatory mediators such as TLR ligands. Here we investigated the contribution of STAT1-independent pathway to macrophage activation and its underlying mechanism in the context of combined stimulation of IFN and TLR. We found that TLR-induced production of inflammatory cytokines (TNF-α, IL-12) was not simply nullified but was significantly suppressed by signaling common to IFN-γ and IFN-β in STAT1-null macrophages. Such a shift in the suppression of TLR response correlated with a sustained STAT3 activation and attenuation of NF-κB signaling. Using a JAK2/STAT3 pathway inhibitor or STAT3-specific siRNA, blocking STAT3 in that context restored TNF-α production and NF-κB signaling, thus indicating a functional cross-regulation among STAT1, STAT3, and NF-κB. Our results suggest that STAT1 deficiency reprograms IFN signaling from priming toward suppression of TLR response via feedback regulation of STAT3, which may provide a new insight into the host defense response against microbial pathogens in a situation of STAT1 deficiency.

Macrophages serve as the first line of defense against microbial pathogens and are vital for the regulation of inflammation^1^,^2^. Macrophages detect and eliminate invading pathogens via pattern recognition receptors (PRRs) that have evolved to sense conserved microbial components, termed pathogen-associated molecular patterns (PAMPs). Toll-like receptors (TLRs) are prototypical and are the best characterized PRR family, which recognize a diverse range of PAMPs such as lipopeptides, lipopolysaccharide (LPS), and viral nucleic acids^3^,^4^,^5^. Upon stimulation, individual TLR recruits different adapter proteins and elicits distinct signaling pathways, including conserved activation of nuclear factor-κB (NF-κB) and mitogen-activated protein kinases (MAPKs), driving a context-dependent immune response^6^,^7^. A prominent manifestation of macrophage activation upon TLR ligation is the production of various inflammatory cytokines including the tumor necrosis factor (TNF)-α, interleukin (IL)-6, IL-12, and interferons (IFNs)^6^. Moreover, macrophage activation is finely controlled by cytokines derived from macrophages or surrounding cells in a way to achieve pathogen clearance but to avoid excessive inflammation causing local tissue damage^8^. Thus, appropriate regulation of macrophage function requires the integration of signals from multiple inflammatory mediators, including exogenous PAMPs and endogenous cytokines.

IFNs are key regulators of macrophage activation and principally trigger signaling of the Janus kinase (JAK)/signal transducer and activators of transcription (STAT) pathway^9^,^10^,^11^. They are classified as type I (IFN-α/β), type II (IFN-γ), and type III (IFN-λs) IFNs. IFN-γ, originally called macrophage-activating factor, predominantly induces the activation and homodimerization of STAT1 for target gene transcription^12^. In contrast, IFN-α/β/λ signaling mainly leads to heterodimerization of STAT1 and STAT2 and their interaction with IFN regulatory factor 9 to the ISG factor 3 transcription factor complex^13^,^14^. The crucial role of STAT1 in IFN-γ and IFN-α/β signaling is well recognized by studies using STAT1-null mice^15^,^16^ and the mutant cell lines^13^. Mice deficient in STAT1 are highly susceptible to infection by both viral and microbial pathogens and tumor formation. Such susceptibility has been attributed to compromised innate immunity largely resulting from the combined loss of IFN responses^16^. Studies of germline human STAT1 mutation also revealed that subjects with complete STAT1 deficiency display overt susceptibility to viral and mycobacterial infections^17^,^18^. These patients are characterized by abrogation of STAT1-dependent responses to both IFN-γ and IFN-α.

Although STAT1-dependent pathways are crucial for promoting immune responses, there is compelling evidences regarding the existence of STAT1-independent pathways in the regulation of gene expression and cellular responses by IFNs, particularly IFN-γ^19^. IFN-γ induces a number of genes in STAT1-null bone marrow-derived macrophages (BMMs); the expression of some genes (e.g. MCP-1, PIM-1) is induced in both wild-type and STAT1-null BMMs irrespective of STAT1 deficiency, whereas some (IL-1β, arginase) are induced selectively in STAT1-null BMMs^19^,^20^. Whereas IFN-γ treatment of diverse cell types leads to the inhibition of cell growth or death in wild-type cells, the same treatment enhances the survival and proliferation of STAT1-null cells^20^,^21^,^22^. Moreover, studies of viral infection in STAT1-null mice revealed that IFN-dependent, STAT1-independent pathways mediate some and fail-safe antiviral responses^20^,^23^,^24^. Therefore, biological consequences of IFN-induced STAT1-independent pathways appear to be diverse (with alternative, opposite, or compensatory outcomes) and context-dependent according to the types of cells and stimuli.

Many functions of IFNs rely on cross-regulation of cellular responses to exogenous inflammatory mediators such as TLR ligands^10^,^25^, a situation that likely occurs during the course of infection. In this context, to better understand the importance of STAT1-independent IFN-signaling pathway in macrophage activation, we explored the functional consequences and underlying mechanism of cross-talk between IFN receptor and TLR in STAT1-null macrophages. The results showed that, in the absence of STAT1, TLR-induced production of inflammatory cytokines (TNF-α, IL-12) is not simply nullified but unexpectedly suppressed by signaling through IFNs (IFN-γ, IFN-β) in the macrophages. Such STAT1-independent suppression of TLR response by IFNs was associated with an intrinsic cell-autonomous mechanism involving a delayed activation of STAT3 and an attenuation of NF-κB signaling. Therefore, this study reveals that STAT1 deficiency redirects the nature of IFN signaling from “priming” to “suppression” of TLR-induced macrophage activation via feedback regulation of STAT3.

## Results

### STAT1 deficiency shifts IFN-γ signaling pathway toward a suppression of TLR response

**Figure 1:**
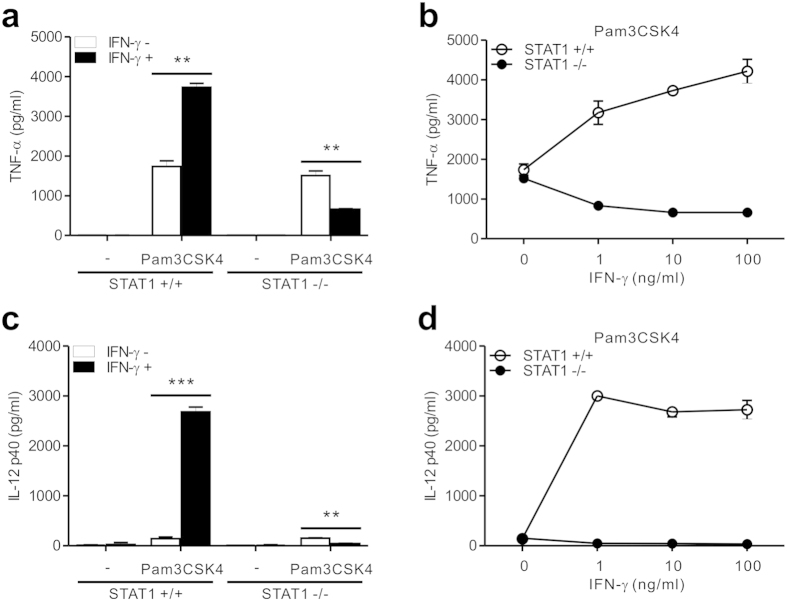
STAT1 deficiency redirects IFN-γ signaling toward a suppression of TLR2-induced TNF-α and IL-12 production. (**a**,**c**) Peritoneal macrophages were left untreated (−) or were treated with Pam3CSK4 (100 ng/ml) for 8 h with or without IFN-γ (10 ng/ml). The production of TNF-α (**a**) and IL-12 p40 (**c**) in the supernatant was determined by ELISA. (**b**,**d**) Macrophages were incubated with Pam3CSK4 (100 ng/ml) with increasing doses of IFN-γ for 8 h. The secretion of TNF-α (**b**) and IL-12 p40 (**d**) in the supernatant was then determined by ELISA. Data represent the means ± SD of three independent experiments. **P < .01 and ***P < .001.

To characterize the inflammatory response of STAT1-null macrophages, we first assessed the effect of IFN-γ on the production of inflammatory cytokines by TLR-stimulated peritoneal macrophages. It has been well appreciated that IFN-γ primes macrophages for enhanced responses to TLR ligands and greatly augments TLR-induced expression of inflammatory cytokines such as TNF-α and IL-12^9^,^10^. Because TLR2 stimulation is linked to the production of TNF-α and IL-12 but not of type I IFNs^26^,^27^, Pam3CSK4, a ligand for TLR2/1, was employed as a standard stimulus to study macrophage responses to IFN-γ. After stimulation with Pam3CSK4 alone, comparable levels of TNF-α were produced from wild-type and STAT1-null macrophages (Fig. 1a). While IFN-γ co-treatment significantly enhanced the production of TNF-α by wild-type macrophages, it unexpectedly and notably suppressed TNF-α production by STAT1-null macrophages (Fig. 1a). To further characterize this opposite response of TNF-α production, we treated STAT1-null macrophages with various doses of IFN-γ (1–100 ng/ml). In contrast to the augmented response of wild-type macrophages, Pam3CSK4-treated STAT1-null macrophages reduced the production of TNF-α by IFN-γ treatment in a dose-dependent manner (Fig. 1b). Moreover, similar and dose-dependent suppression of IL-12 production was observed in STAT1-null macrophages treated with IFN-γ/Pam3CSK4 combination (Fig. 1c,d). This was in contrast to the substantial increase of IL-12 production by wild-type macrophages in response to the same treatment. Thus, IFN-γ appears to positively or negatively regulate Pam3CSK4-induced production of inflammatory cytokines such as TNF-α and IL-12 depending on the presence of STAT1.

**Figure 2:**
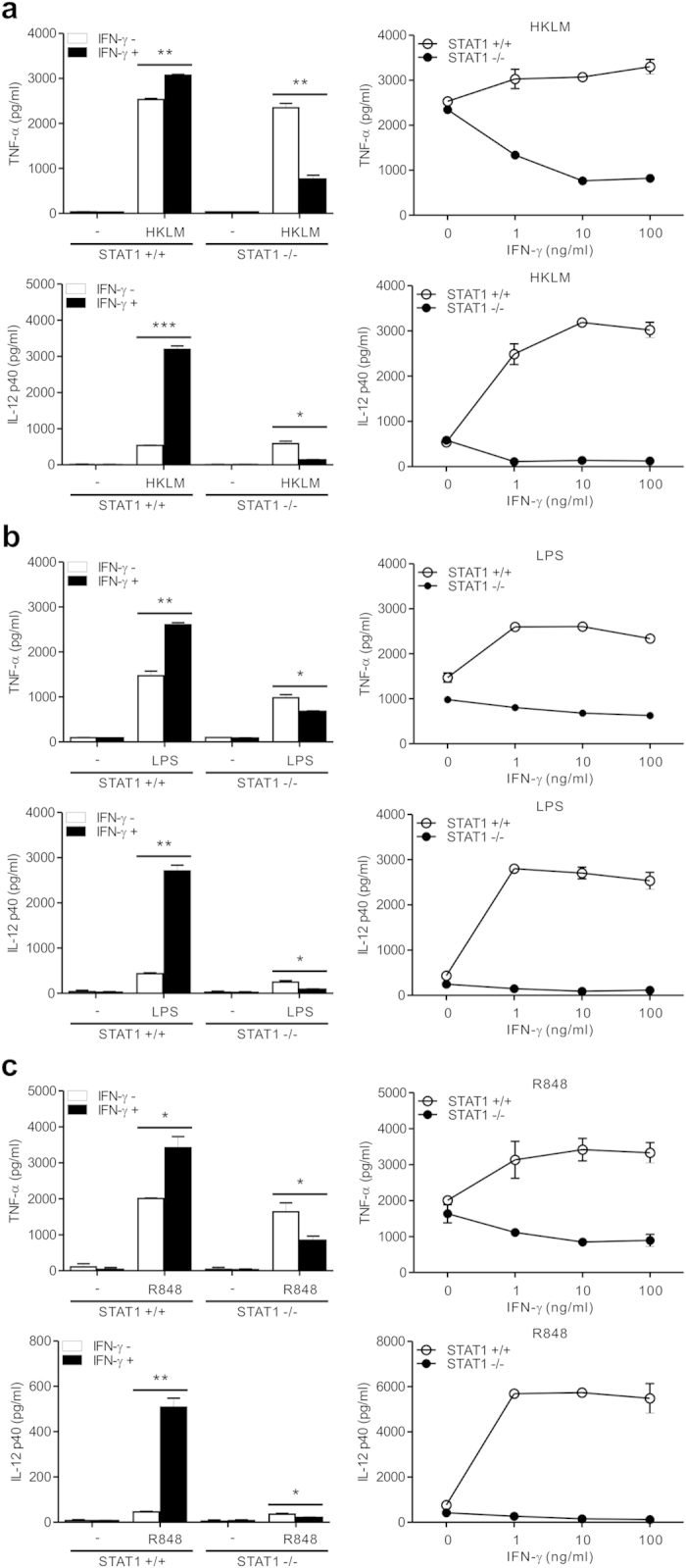
IFN-γ-induced suppression of TNF-α and IL-12 production by STAT1 deficiency is a common feature to stimulation via diverse TLRs. Macrophages were incubated with HKLM (10^8^ cells/ml) (**a**) LPS (100 ng/ml) (**b**) or R848 (1 μg/ml) (**c**) for 8 h with 10 ng/ml of IFN-γ (*left panel*) or increasing doses of IFN-γ (*right panel*). The production of TNF-α (*top panel*) and IL-12 p40 (*bottom panel*) in the supernatant was determined by ELISA. Data represent the means ± SD of three independent experiments. *P < .05, **P < .01 and ***P < .001.

To determine whether such a dichotomous response to IFN-γ for cytokine production was attributed to specific TLR, STAT1-null macrophages were treated with diverse PAMPs that engage different TLRs. We found a similar shift of IFN-γ pathway toward a suppression of TNF-α and IL-12 production by STAT1-null macrophages treated with HKLM (Heat Killed *Listeria monocytogenes*) for TLR2 (Fig. 2a), LPS for TLR4 (Fig. 2b), R848 for TLR7/8 (Fig. 2c), and CpG ODN1826 for TLR9 (Supplementary Fig. S1a). Thus, these results suggest that the repression of inflammatory cytokine production by IFN-γ in the absence of STAT1 is not limited to specific TLR stimulation but is likely to be a common feature to stimulation through diverse TLRs. We next investigated whether the repression of inflammatory cytokine production by IFN-γ in STAT1-null macrophages could be attributed to a defect in the expression of the IFN-γ receptor. The expression levels of signal-transducing subunit (IFN-γR2) were comparable between the wild-type and STAT1-null macrophages (Supplementary Fig. S2), suggesting that such a shift of IFN-γ pathway toward a suppression of TLR-response is likely to occur at a step downstream of IFN-γ receptor activation.

### IFN-β suppresses TLR-induced inflammatory cytokine production in the absence of STAT1

**Figure 3:**
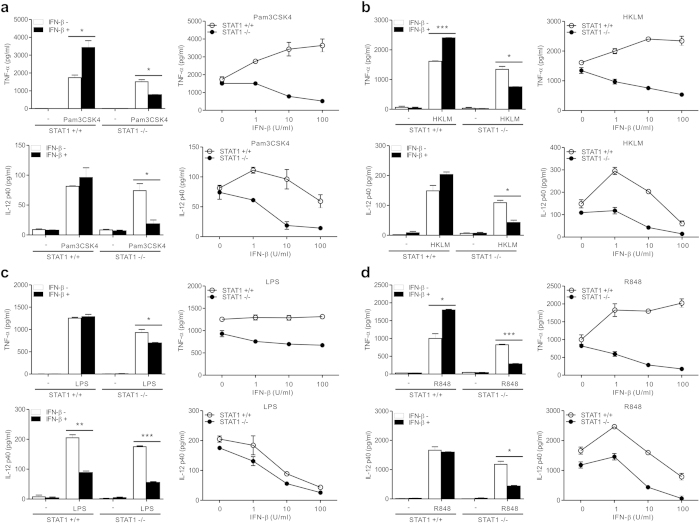
IFN-β suppresses TLR-induced production of TNF-α and IL-12 by STAT1 deficiency. Macrophages were incubated with Pam3CSK4 (100 ng/ml) (**a**) HKLM (10^8^ cells/ml) (**b**) LPS (100 ng/ml) (**c**) or R848 (1 μg/ml) (**d**) for 8 h with 10 U/ml of IFN-β (*left panel*) or increasing doses of IFN-β (*right panel*). The production of TNF-α (*top panel*) and IL-12 p40 (*bottom panel*) in the supernatant was determined by ELISA. Data represent the means ± SD of three independent experiments. *P < .05, **P < .01 and ***P < .001.

STAT1 is the primary signaling mediator for type I as well as type II IFNs^9^,^11^. Moreover, IFN-β has been shown to potentiate TNF-α expression in macrophages stimulated with various TLR ligands, whereas it can negatively regulate IL-12 expression in a manner dependent on IL-10 production^28^,^29^,^30^. Thus, we studied whether STAT1 deficiency also redirects IFN-β pathway into suppressing TLR-induced inflammatory cytokine production. IFN-β significantly and dose-dependently augmented TNF-α production from wild-type macrophages treated with Pam3CSK4 (Fig. 3a, top panel), HKLM (Fig. 3b, top panel), R848 (Fig. 3d, top panel), and CpG ODN1826 (Supplementary Fig. S1b). IFN-β in combination with LPS did not enhance TNF-α production (Fig. 3c, top panel), an observation compatible with that of a previous study^30^. In contrast, TNF-α production from STAT1-null macrophages induced by TLR ligands tested was significantly and dose-dependently suppressed after IFN-β treatment (Fig. 3, top panel). Likewise, IFN-β significantly decreased the TLR-induced production of IL-12 by STAT1-deficiency (Fig. 3, bottom panel). Consistent with a negative regulation of IL-12 expression by type I IFNs^31^, IFN-β could not augment TLR-induced IL-12 production by wild-type macrophages (Fig. 3, bottom panel). Thus, these results suggest that, in the absence of STAT1, STAT1-independent pathway common to IFN-γ and IFN-β stimulation mediates the suppression of TLR-induced inflammatory cytokine production.

### IFNs suppress the transcription of cytokine genes by STAT1 deficiency

**Figure 4:**
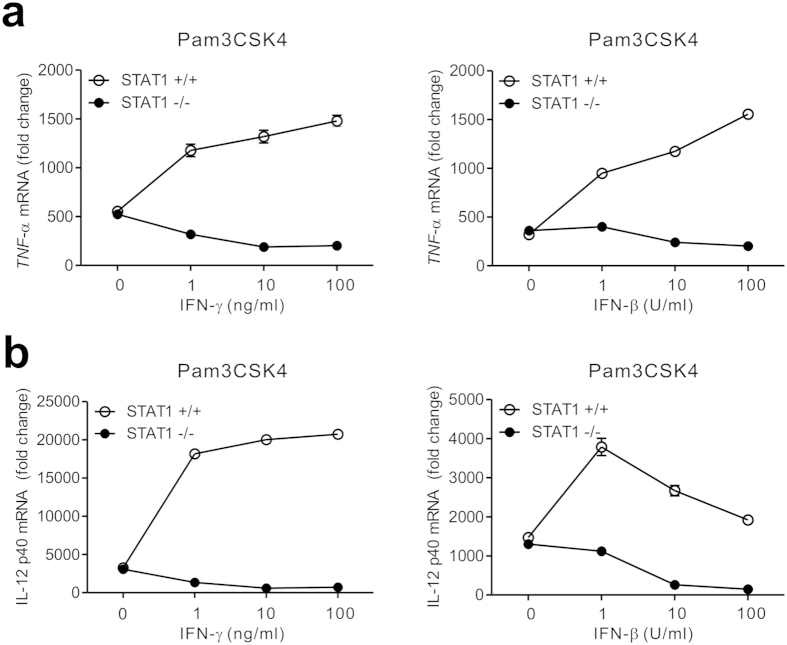
IFNs suppress the gene transcription of TNF-α and IL-12 by STAT1 deficiency. Macrophages were incubated with Pam3CSK4 (100 ng/ml) for 4 h with increasing doses of IFN-γ (*left panel*) and IFN-β (*right panel*). The relative mRNA levels of TNF-α (**a**) and IL-12 p40 (**b**) were determined by quantitative real-time PCR and normalized to GAPDH mRNA. Data are representative of three independent experiments and are presented as fold change relative to unstimulated cells.

To gain an insight into the underlying mechanism, we next investigated whether the dichotomous response to IFNs for cytokine production could occur at the level of gene expression. By using quantitative real-time PCR (qPCR), we observed that both IFN-γ and IFN-β mediate dose-dependent suppression in the mRNA expression of TNF-α and IL-12 in Pam3CSK4-treated STAT1-null macrophages; this suppression was abrogated or reversed in the presence of STAT1 (Fig. 4). Thus, the similar patterns of cytokine expression and production in response to IFNs suggest an involvement of transcription-dependent mechanism in the STAT1-independent suppression of TLR response.

### Altered regulation of NF-κB and STAT3 signaling in response to IFNs by STAT1 deficiency

**Figure 5:**
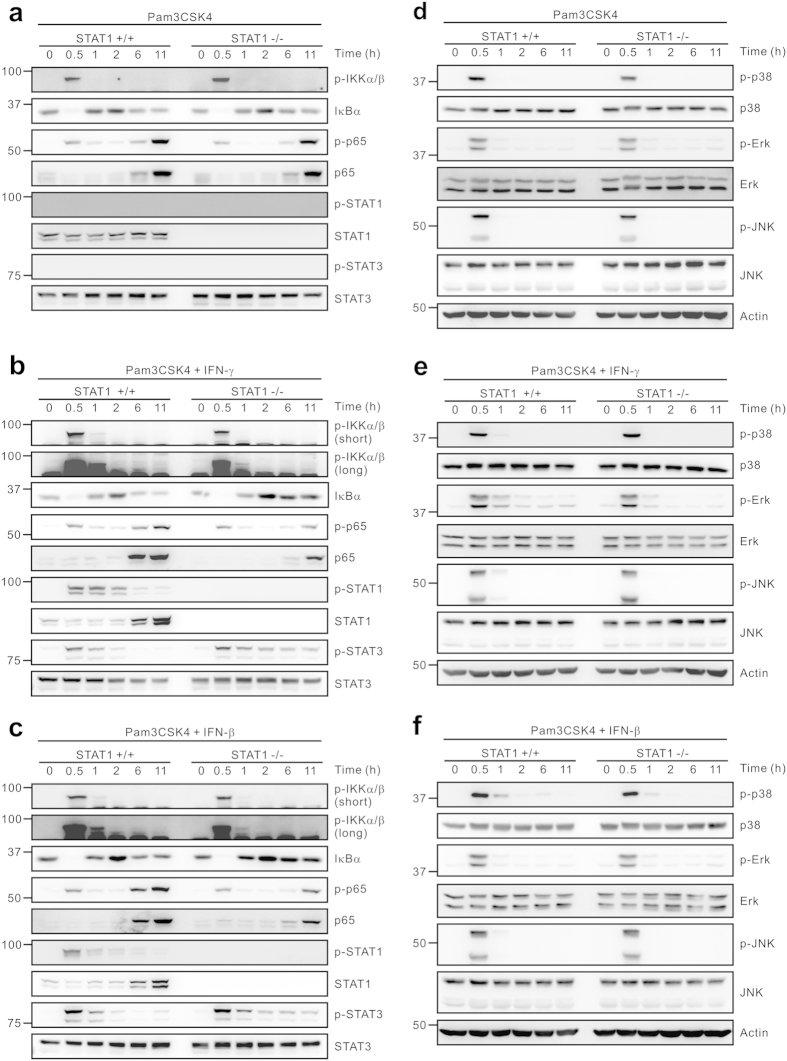
NF-κB and STAT3, but not MAPKs, are dysregulated by IFNs in STAT1-null macrophages. Macrophages were incubated with Pam3CSK4 (100 ng/ml) for the indicated times in the absence (**a**,**d**) or the presence of 10 ng/ml IFN-γ (**b**,**e**) or 10 U/ml IFN-β (**c**,**f**). Cells were then lysed and the lysates were immunoblotted with antibodies specific for the indicated proteins. The assays were run under the same experimental conditions and the blots were cropped from original full-sized images (attached as supplemental Fig. S8). Data are representative of at least three independent experiments.

To study the mechanism of redirected suppression of TLR response by IFNs, we next assessed the signaling pathway triggered by TLR in combination with IFNs. Because such a suppression of TLR response was independent of TLR ligands and IFNs tested, we focused on the activation of signaling molecules common to different TLRs and IFNs. The conserved pathway induced by most TLRs involves the activation of the transcription factor NF-κB and the MAPKs, both of which play a critical role in the regulation of gene transcription for cytokine production^6^. Pam3CSK4 alone induced similar levels of NF-κB activation between wild-type and STAT1-null macrophages, as demonstrated by IKKα/β phosphorylation, IκBα degradation, and the phosphorylation and the level of NF-κB p65 subunit (Fig. 5a). The levels of MAPK activation such as phosphorylation of p38 MAPK, Erk, and JNK were also comparable between wild-type and STAT1-null macrophages stimulated with Pam3CSK4 alone (Fig. 5d). Of note, after co-treatment with IFN-γ or IFN-β, the levels of NF-κB activation were similar or slightly attenuated at early time points (0.5, 1 h) but were clearly impaired at late time points (6, 11 h) by STAT1 deficiency (Fig. 5b,c). However, there was no significant difference in the levels of MAPK activation between wild-type and STAT1-null macrophages by the same treatment (Fig. 5e,f). Thus, these results suggest a dysregulation of NF-κB signaling as a potential mechanism of redirected suppression of TLR response.

Both IFN-γ and IFN-β can induce the recruitment and phosphorylation of STAT1 and STAT3, which in turn leads to the formation of their respective homodimers for the regulation of cytokine expression^11^. Pam3CSK4 alone was unable to induce the phosphorylation of STAT1 and STAT3 at all time points examined (Fig. 5a). Of note, in the absence of STAT1, STAT3 phosphorylation was sustained in response to both IFN-γ and IFN-β treatment, which was pronounced at late time points (6, 11 h) (Fig. 5b,c). In comparison, the same treatment did not affect the levels of STAT3 expression in both wild-type and STAT1-null macrophages. Although dysregulation of other signaling molecules cannot be excluded, our results suggest that the dysregulation of NF-κB and/or STAT3 by IFN-induced STAT1-independent pathway may contribute to the redirected suppression of TLR-response.

### Association of NF-κB attenuation with STAT3 activation in STAT1-null macrophages

Given the inverse correlation between NF-κB and STAT3 activation at late time points, we next investigated whether the regulation of NF-κB and STAT3 was interconnected in STAT1-null macrophages. By using immunofluorescence staining and confocal microscopy, we determined the localization of p65 and phospho-STAT3 in macrophages stimulated with Pam3CSK4 in the presence or absence of IFN-γ. As shown in Supplementary Fig. S3, a robust translocation of p65 to the nucleus was detected at an early (0.5 h) and late (6 h) time point in both macrophages treated with Pam3CSK4. After co-treatment with IFN-γ, co-localization of phospho-STAT3 and p65 in the nucleus was frequently observed at early time point. In comparison, the levels of phospho-STAT3 in the nucleus were sustained at late time point (6 h) in STAT1-null macrophages, which correlated with the decrease of nuclear p65 levels, in line with the results of immunoblotting. It has been shown that STAT3 can negatively regulate the activation of NF-κB in normal immune cells^32^,^33^. To determine whether this is the case in STAT1-null macrophages, we used a selective inhibitor of the JAK2/STAT3 pathway, pyrazolyl pyrimidine AZD1480^34^. Macrophages were stimulated with IFN-γ and/or Pam3CSK4 in the presence of increasing doses of AZD1480 (1–10 μM). Intriguingly, AZD1480 was capable of restoring the IFN-γ-mediated suppression of TNF-α production from Pam3CSK4-stimulated STAT1-null macrophages (Supplementary Fig. S4). AZD1480 also dose-dependently inhibited TNF-α production from wild-type macrophages treated with IFN-γ/Pam3CSK4 combination, probably due to an inhibition of JAK2/STAT1 pathway. Moreover, a separate time course study on cytokine production showed that TNF-α production by STAT1-null macrophages was upregulated in a time-dependent manner in response to Pam3CSK4 alone but was repressed by co-treatment with IFN-γ and IFN-β (Supplementary Fig. S5). Such suppressive effects on TLR response by IFNs became apparent at late time points (over 6 h), thus supporting a potential link between IFN-induced suppression of TLR response and sustained activation of STAT3 by STAT1 deficiency.

**Figure 6:**
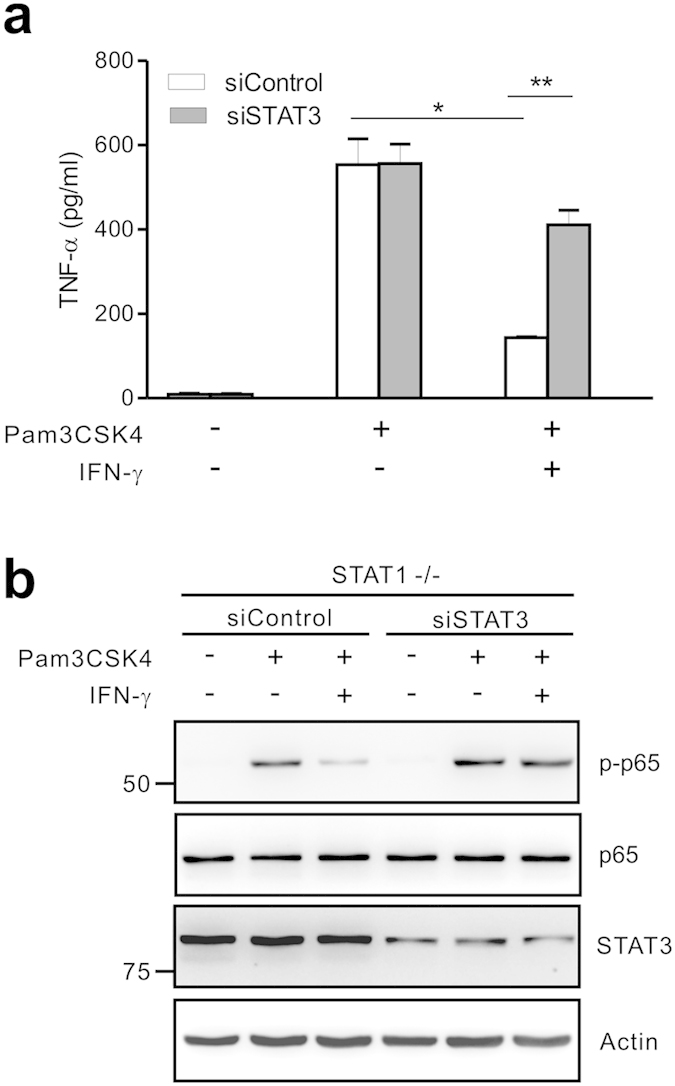
Combined STAT3 deficiency reverses defective TNF-α production and NF-κB signaling. (**a**) STAT1-null BMMs were transfected with control siRNA or siRNA specific for STAT3. Cells were then incubated with Pam3CSK4 (100 ng/ml) for 8 h with or without IFN-γ (10 ng/ml). The production of TNF-α in the supernatant was determined by ELISA. (**b**) STAT1-null BMMs that were transfected with control siRNA or STAT3 siRNA were stimulated with Pam3CSK4 (100 ng/ml) for 11 h with or without IFN-γ (10 ng/ml). Cell lysates were then immunoblotted with antibodies specific for the indicated proteins. The assays were run under the same experimental conditions and the blots were cropped from original full-sized images (attached as supplemental Fig. S8). Data represent the means ± SD of three independent experiments. *P < .05 and **P < .01.

Based on this promising result, we performed knockdown of STAT3 by small interfering RNA (siRNA) in STAT1-null macrophages to directly probe the role of STAT3 in such context. BMMs were prepared from STAT1-null mice and then transfected with siRNA specific for STAT3, which resulted in a noticeable reduction in the amount of STAT3 in the cells after 48 h. Of note, IFN-γ suppressed TNF-α production from Pam3CSK4-stimulated STAT1-null BMMs, which was restored significantly by the knockdown of STAT3 (Fig. 6a). Moreover, STAT3 knockdown also restored p65 phosphorylation that was attenuated by IFN-γ-induced STAT1-independent pathway (Fig. 6b). Thus, these results suggest a functional relationship between STAT3 and NF-κB and that IFN-induced sustained activation of STAT3 in the absence of STAT1 contributes to the attenuation of NF-κB activity.

### Sustained STAT3 activation by IFNs relies on a cell-autonomous mechanism

**Figure 7:**
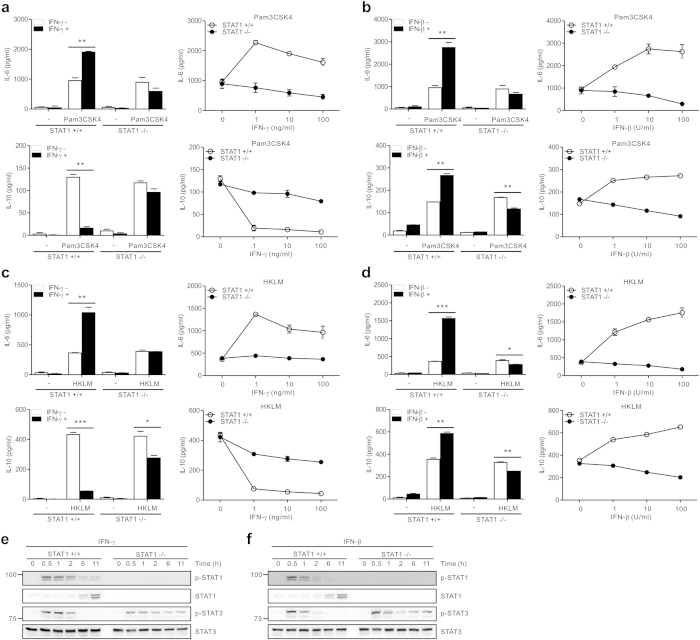
Sustained STAT3 activation relies on cell-intrinsic signaling triggered by IFNs. (**a**,**b**) Macrophages were incubated with Pam3CSK4 (100 ng/ml) for 8 h with IFN-γ (**a**) or IFN-β (**b**). The production of IL-6 (*top panel*) and IL-10 (*bottom panel*) in the supernatant was determined by ELISA. (**c**,**d**) Macrophages were incubated with HKLM (10^8^ cells/ml) for 8 h with IFN-γ (**c**) or IFN-β (**d**). The production of IL-6 (*top panel*) and IL-10 (*bottom panel*) in the supernatant was then determined. (**e**,**f**) Macrophages were incubated with 10 ng/ml IFN-γ (**e**) or 10 U/ml IFN-β (**f**) for the indicated times. Thereafter, cells lysates were immunoblotted with antibodies specific for the indicated proteins. The assays were run under the same experimental conditions and the blots were cropped from original full-sized images (attached as supplemental Fig. S8). Data represent the means ± SD of three independent experiments. *P < .05, **P < .01 and ***P < .001.

Next, we sought to identify the mechanism underlying sustained STAT3 activation by IFNs in STAT1-null macrophages. Activated macrophages stimulated with Pam3CSK4 can produce a significant level of cytokines including IL-6 and IL-10 that primarily signal via STAT3^35^,^36^. In this respect, we assessed the production of IL-6 and IL-10 from Pam3CSK4-treated macrophages combined with IFNs to determine whether sustained STAT3 activation might be accounted for by their dysregulated production. Although both IFN-γ and IFN-β augmented IL-6 production from activated wild-type macrophages, they slightly decreased IL-6 production by STAT1 deficiency (Fig. 7a,b). In addition, in the absence of STAT1, IL-10 production was also slightly reduced by both IFN-γ and IFN-β in a dose-dependent manner (Fig. 7a,b). In comparison, in the presence of STAT1, IL-10 production was suppressed by IFN-γ but was augmented by IFN-β, consistent with the results of previous studies^37^,^38^. Given that the amounts of IL-6 and IL-10 by Pam3CSK4 alone-treated macrophages was unable to induce STAT3 phosphorylation (Fig. 5a), it is highly unlikely that sustained phosphorylation of STAT3 in STAT1-null macrophages involved dysregulated production of IL-6 and/or IL-10. Similar patterns of IL-6 and IL-10 production were also observed when macrophages were stimulated with HKLM, another TLR2 ligand, instead of Pam3CSK4 (Fig. 7c,d).

Thus, we hypothesized that the sustained STAT3 activation by STAT1 deficiency could be mediated by cell-intrinsic signaling pathway independently of cytokines released from macrophages. It has been shown that both IFN-γ and IFN-β are independently capable of inducing the phosphorylation of STAT3 as well as STAT1^11^. When we exposed macrophages to IFN-γ or IFN-β alone, sustained phosphorylation of STAT3 was again observed in STAT1-null macrophages (Fig. 7e,f), similar to the results of its combination with Pam3CSK4. Moreover, the levels of phospho-STAT3 in the nucleus were sustained by treatment with IFN-γ alone in STAT1-null macrophages (Supplementary Fig. S3). Therefore, these data suggest that sustained STAT3 activation required for NF-κB attenuation is likely due to cell-intrinsic signaling triggered by IFN-γ and IFN-β, rather than by cytokines released from activated macrophages. Supporting this, the production of various cytokines including IL-6 and IL-10 by IFNs alone was negligible in both macrophages (Figs 1, 2, 3 and 7).

## Discussion

It has been long appreciated that IFN-induced signaling typically augments TLR-triggered macrophage activation through a STAT1. In this study, we offer a new perspective on the signaling pathway activated by IFN-γ and IFN-β, which unexpectedly suppresses TLR-induced production of inflammatory cytokines (TNF-α, IL-12) by STAT1 deficiency. Using a model of macrophage activation through diverse TLRs that couple to different signaling adaptors, we found that such a suppression of TLR response was independent of TLR ligands and IFNs tested. Among the conserved signaling pathways of TLRs, the activation of NF-κB, a key transcription factor for cytokine expression, but not of MAPK was significantly attenuated during the suppression. Of interest, in the absence of STAT1, both IFN-γ and IFN-β induced sustained phosphorylation of STAT3 in a cell-autonomous fashion, and the depletion of STAT3 in that context significantly restored TNF-α production and NF-κB activation from STAT1-independent suppression of the TLR response. Thus, our results suggest an unexpected cross-regulation among STAT1, STAT3, and NF-κB in the context of combined IFN and TLR stimulation where STAT1 deficiency tipped the homeostatic balance toward a suppression of the TLR response via deregulated STAT3 activation.

A number of studies have shown that STAT1 and STAT3 can cross-regulate each other and often play opposing roles in diverse biological processes such as proliferation, apoptosis, and inflammation^39^,^40^,^41^. For example, STAT1 activation induces growth arrest and promotes apoptosis, whereas activated STAT3 can protect cells from apoptosis and promote proliferation through the induction of certain anti-apoptotic genes and oncogenes^39^. In addition, STAT1 activation via IFNs typically mediates a pro-inflammatory response that favors the recruitment and activation of immune cells including macrophages to the inflamed site^41^. By contrast, STAT3 plays a key role in anti-inflammatory effects of IL-10 on macrophages and dendritic cells in part by down-regulating STAT1 activation^42^. Besides the functional antagonism between STAT1 and STAT3 in many biological processes, they can also reciprocally regulate each other’s activation^43^,^44^. IFN-γ stimulates a predominant activation of STAT1 and an additional activation of STAT3 to a lesser extent/more transiently. In STAT1-null mouse embryonic fibroblasts (MEFs), IFN-γ induces stronger and prolonged activation of STAT3 and transcription of its target genes^43^, suggesting a STAT1-dependent down-regulation of STAT3. Similarly, in STAT3-null MEFs, gp130 cytokine IL-6 induces prolonged STAT1 activation and STAT1-dependent genes, thereby triggering an IFN-γ-like response^44^. Usually, macrophages become activated for cytokine production in response to IFN-γ, along with the exposure to exogenous PAMPs such as LPS^45^,^46^. IFN-γ plays a priming role but does not activate macrophages on its own^46^. In this respect, it has been obscure how the cross-regulation between STAT1 and STAT3 regulate cellular responses to exogenous inflammatory mediators such as TLR ligands. In this study, we found that macrophage inflammatory responses in the context of diverse TLR stimulation could be regulated by a homeostatic cross-regulation between STAT1 and STAT3 besides its previously appreciated role in the maintenance of cytokine signaling specificity (e.g. IFN-γ/STAT1 vs. IL-6/STAT3)^43^,^44^.

Among the conserved signaling pathways triggered by diverse TLRs, activation of NF-κB but not of MAPKs was primarily affected by STAT1 deficiency through deregulated and sustained activation of STAT3. Thus, inhibition of NF-κB signaling may account for one potential mechanism underlying anti-inflammatory effect associated with STAT3 activation in macrophages. It has been demonstrated a functional crosstalk between STAT3 and NF-κB, including a direct interaction between STAT3 and a component of NF-κB^32^,^33^,^47^,^48^. These studies revealed that STAT3 modulation could significantly but not uniformly influence NF-κB activation depending on the types of stimuli and cells. STAT3 has been implicated in the down-regulation of stimulus (e.g. LPS, TNF-α)-induced NF-κB activation in dendritic cells and tumor cells^32^,^48^. In contrast, STAT3 is required to maintain constitutive NF-κB RelA activity in tumor cells^48^. STAT3 was also shown to activate noncanonical NF-κB pathway in breast cancer cells and breast cancer-derived myeloid-derived suppressor cells^49^,^50^. These studies suggest that NF-κB activation affected by STAT3 appear to be diverse and context-dependent. In this study, we provide evidence in primary macrophages that unrestrained STAT3 signaling due to STAT1 deficiency negatively regulates TLR-induced NF-κB activation and inflammatory cytokine production. This notion was supported by the finding that targeted inhibition of STAT3 in the context of STAT3 deregulation restored TLR2-induced TNF-α production and NF-κB signaling.

Sustained STAT3 activation was induced in STAT1-null macrophages through signaling common to IFN-γ and IFN-β irrespective of TLR stimulation. STAT3 is a predominant signaling mediator of IL-6 and IL-10^35^,^36^. TLR2 ligation alone that is linked to the production of IL-6 and IL-10 but not type I IFNs did not cause STAT3 phosphorylation in STAT1-null macrophages, suggesting that the amount of IL-6 and IL-10 released might be insufficient. Further, co-treatment with both IFN-γ and IFN-β only slightly decreased the production of IL-6 and IL-10 by STAT1 deficiency. Thus, it is unlikely that deregulated STAT3 activation is mediated by inflammatory cytokines including IL-6 and IL-10 released from TLR-activated macrophages. Instead, the stimulation of STAT1-null macrophages with IFN-γ or IFN-β alone not inducing a significant amount of IL-6 and IL-10 could lead to the prolonged phosphorylation of STAT3, suggesting the primary role of cell-intrinsic IFN signaling in such a context. Further, the unrestrained STAT3 activation was pronounced at late time points (~6 h) rather than at early time points in STAT1-null macrophages. Based on the delayed kinetics of STAT3 activation, we speculate that the cross-regulation between STAT1 and STAT3 is regulated by an indirect feedback mechanism involving transcriptional induction of inhibitory molecules that oppose each other among several proposed mechanisms^10^,^51^,^52^. In this respect, the sustained STAT3 activation by STAT1 deficiency might be accounted for by a defective transcription of the inhibitory molecules suppressing STAT3 activation. Of interest, the delayed STAT3 activation in macrophages was distinct from the rapid kinetics (~15 min) of STAT3 activation observed in STAT1-null MEF treated with IFN-γ^43^, suggesting the possibility of cell-type specific regulation of opposing STATs. Although further studies are required to clarify the underlying mechanism, our results suggest an important role for cross-regulation among STAT1, STAT3, and NF-κB in directing the macrophage response to multiple activation cues, such as type I or II IFN combined with diverse TLR ligands.

Although we suggest deregulated STAT3 as a potential mechanism that mediates the suppression of TLR response by STAT1 deficiency, the involvement of other regulatory mechanism(s) in such redirected suppression cannot be excluded. It is possible that certain negative regulators for TLR signaling are upregulated by TLR and/or IFN stimulation and contribute to the suppression of TLR response. Thus, we assessed at different time points the expression levels of several negative regulators for TLR signaling, which include SOCS1/3, Triad3a, SHP-1, A20, and Pin1^53^. It revealed that the expression of SOCS3 but not of others is significantly augmented by combined IFN-γ and TLR2 stimulation in STAT1-null macrophages compared to wild-type macrophages (Supplementary Fig. S6). Moreover, such an increase in SOCS3 expression was pronounced at late time points (6, 10 h), which coincided with sustained STAT3 activation. Together with a previous report of SOCS3 as a STAT3-target gene^54^, our result suggests a possibility that deregulated STAT3 activation may contribute to the redirected suppression of TLR response at least in part via SOCS3 upregulation. Given numerous negative regulators for TLR signaling^53^, further studies are required to determine the contribution of other negative regulator(s) including SOCS3 in IFN-induced suppression of TLR response by STAT1 deficiency.

In addition, we assessed whether similar suppression of inflammatory cytokine production is observed in STAT1-null macrophages treated with IFN-γ and non-TLR agonist combination. In contrast to the augmented response of wild-type macrophages, we found a similar shift of IFN-γ pathway toward a suppression of TNF-α production by STAT1-null macrophages in response to the transfection with poly(I:C), plasmid DNA, and interferon stimulatory DNA (ISD) (Supplementary Fig. S7), which engage different cytoplasmic nucleic acid sensors^55^. Although the degree to which IFN-γ suppressed TNF-α production by STAT1 deficiency varied by stimuli, it was significantly observed. Thus, such a dichotomous response to IFN-γ for TNF-α production suggests a possibility that STAT1-independent suppression of inflammatory response by IFN-γ is not limited to TLR-mediated stimulation but appears to be context-dependent according to the types of stimuli. In this respect, further studies are required to determine the specificity of STAT1-independent suppression of inflammatory response in the context of other stimuli and underlying mechanism involved.

Functional STAT1 is critical to host defense against microbial infection. STAT1 deficiency in mice and its loss-of-function mutations in humans have been causally linked to diverse infectious diseases. Individuals with complete STAT1 deficiency developed fatal viral and mycobacterial infections^17^,^56^. STAT1 can be targeted by certain viruses that cause its depletion or functional abrogation^57^,^58^. Thus, IFN-induced suppression of the TLR response in the absence of STAT1, as shown here, may occur during the course of microbial infection associated with STAT1 deficiency and/or is more likely in patients with defective expression of STAT1^17^,^56^,^57^,^58^, the functional consequences of which await further investigation.

## Methods

### Animals

C57BL/6 mice were purchased from Jackson Laboratory. STAT1-null mice were from Taconic Farms and were backcrossed to C57BL/6 mice for 12 generations^59^. 8- to 10-week-old C57BL/6 wild-type (STAT1+/+) and age-matched STAT1-null (STAT1−/−) mice were used. All mice were housed and fed under a specific-pathogen-free condition.

### Ethics statement

All animal experiments in this work were approved by Institutional Animal Care and Use Committee (IACUC) of Asan Institute for Life Sciences with license number of 2012-02-074 and done in accordance with the approved guidelines set forth by IACUC.

### Macrophage isolation and culture

Peritoneal macrophages were isolated from 8- to 10-wk-old female mice by peritoneal lavage, 3 days after intraperitoneal injection of 3.85% thioglycolate (Difco), plated on plastic tissue culture plates, and incubated at 37 °C for 3 h. Non-adherent cells were removed by three repeated washings with fresh Dulbecco´s modified Eagle medium (DMEM), and the adherent macrophages were cultured overnight in DMEM supplemented with 10% fetal bovine serum (FBS), 100 U/ml penicillin, 100 μg/ml streptomycin, and 2 mM glutamine. BMMs were prepared from 8- to 10-wk-old female mice as previously described^60^. In brief, bone marrow cells from the femur and tibia were cultured in DMEM containing 10% FBS, 100 U/ml penicillin, 100 μg/ml streptomycin, and 2 mM glutamine for 6 h. Nonadherent cells were harvested and cultured for 6 days in the presence of M-CSF (20 ng/ml) with a medium exchange being made every two days. After culture, BMMs were washed with DPBS and then were used for the indicated assays.

### Reagents and antibodies

Following TLR ligands were obtained from InvivoGen: Pam3CSK4 (TLR1/2), HKLM (TLR2), LPS (TLR4), R848 (TLR7/8), and CpG ODN1826 (TLR9). Non-TLR agonists were also from InvivoGen as follows: Poly(I:C), Poly(dA:dT), and ISD^61^ (a 45-base-pair double-stranded DNA). DNA and RNA transfection was also performed using Lipofectamine LTX (Invitrogen) as previously described^55^. Mouse recombinant IFN-β was purchased from PBL Interferon Source and mouse IFN-γ was from R&D System. AZD1480 (JAK2 inhibitor) was from Selleck chemicals. Mouse recombinant M-CSF was obtained from PeproTech. Antibodies used in this study were listed according to their targets with their sources indicated: pY701-STAT1, STAT1, pY705-STAT3 (D3A7), STAT3, p-IKKα/β (16A6), pS536-NFκB p65 (93H1), p-p38 MAPK, p38 MAPK (D13E1), p-p44/42 MAPK (Erk1/2), p44/42 MAPK (Erk1/2), p-SAPK/JNK (81E11) and SAPK/JNK (56G8) (Cell Signaling); IκB-α (C-21) and NFκB p65 (F-6) (Santa Cruz Biotechnology); Actin (C4, BD Biosciences). Horseradish peroxidase (HRP)-conjugated antibodies against mouse and rabbit antibodies were from Santa Cruz Biotechnology. Alexa Fluor 488-conjugated goat anti-mouse F(ab´)2 and Alexa Fluor 647-conjugated goat anti-rabbit F(ab´)2 for confocal microscopy were obtained from Jackson ImmunoResearch Laboratories.

### ELISA

Supernatants from cultured macrophages were used to determine the amount of TNF-α, IL-6, IL-10, IL-12 p40 released. Enzyme-linked immunosorbent assays (ELISA) were carried out according to the manufacturer’s recommendation (R&D Systems).

### Western blot analysis

Macrophages were lysed in lysis buffer (50 mM Tris-HCl [pH 7.5], 150 mM NaCl, 1% Triton X-100, 5 mM EDTA, 0.5 mM Na_3_VO_4_, 50 mM NaF, 1 mM PMSF, and protease inhibitor cocktail [Thermo]) for 30 min on ice. Cell debris was removed by centrifugation at 20,000 × g for 15 min at 4 °C. Protein concentration in cell lysates was determined using a Bio-Rad protein assay kit. An equal amount of protein for each sample was separated by 8 or 10% SDS-PAGE and subsequently transferred onto PVDF membrane (Millipore). After blocking with 5% skim milk for 1 h in TBS-T (0.1% Tween 20 in TBS), membranes were incubated with primary antibodies and then with the respective HRP-conjugated secondary antibody (Santa Cruz). Blots were developed with SuperSignal West Pico (Pierce) and signals were detected with LAS-4000 (Fujifilm).

### RNA analysis

Total RNAs from macrophages, that had been left or stimulated with Pam3CSK4 with or without IFN (IFN-γ or IFN-β) for the indicated times, were isolated using TRIzol reagent (Invitrogen). cDNA was synthesized from 1 μg RNA using the ReverTra Ace qPCR RT kit (Toyobo) according to manufacturer’s instructions. RNA analysis using real-time PCR was performed as described^62^. The following PCR primers were used: 5′-CCT GTA GCC CAC GTC GTA GC-3′ (Forward) and 5′-TTG ACC TCA GCG CTG AGT TG-3′ (Reverse) for mouse TNF-α; 5′-CAG AAG CTA ACC ATC TCC TGG TTT G-3′ (Forward) and 5′-TCC GGA GTA ATT TGG TGC TTC ACA C-3′ (Reverse) for mouse IL-12 p40; 5′-AGT ATG ATG ACA TCA AGA AGG-3′ (Forward) and 5′-ATG GTA TTC AAG AGA GTA GGG-3′ (Reverse) for mouse GAPDH. Following PCR primer pairs for the detection of negative regulators for TLR signaling were obtained from Qiagen: mouse SOCS1 (PPM05151A), mouse SOCS3 (PPM05161B), mouse Triad3a (Rnf216; PPM41791A), mouse SHP-1 (Ptpn6; PPM36442A), mouse A20 (Tnfaip3; PPM03207A), and mouse Pin1 (PPM04559A).

### RNA interference

BMM cells were transfected with 300 pmoles of siRNA with the Amaxa Nucleofector II system, as previously described^63^, with slight modifications. A total of 5 × 10^6^ cells were resuspended in 100 μl of Amaxa kit solution T (Lonza), mixed with siRNA, and immediately transfected with program T-20. Cells were then incubated for 4 h at 37 °C, and the adherent cells were harvested with Detachin (Genlantis). Thereafter, cells were cultured in the medium containing M-CSF (20 ng/ml) for 48 h at 37 °C, and then the cells were assayed as indicated. The siRNA specific for mouse STAT3 was obtained from Integrated DNA Technologies (IDT). The following siRNA sequences were used: mouse STAT3: 5′-CAGGGUGUCAGAUCACAUGGGCUAA-3′ (sense) and 5′-UUAGCCCAUGUGAUCUGACACCCUGAG-3′ (antisense). The negative siRNA control was obtained from IDT.

### Flow cytometry

Macrophages were washed in DPBS supplemented with 2.5% horse serum. Fc block was done with anti-FcγRII/III monoclonal antibody (Pharmingen) and 10% normal mouse serum. Cells were stained with anti-IFN-γR2 monoclonal antibody (R&D Systems), and then with FITC-conjugated secondary antibody (Zymed) for analysis by Accuri C6 (Becton Dickinson).

### Immunofluorescence and confocal analysis

The cells on coverslips (Marienfeld) were fixed with 4% paraformaldehyde (Electron Microscopy Sciences) in PBS for 30 min at room temperature (RT) and in cold methanol for 10 min at −20 °C. Then, cells were permeabilized with 0.1% Triton X-100 (Sigma) and 0.1% sodium citrate (Sigma) in PBS for 3 min at 4 °C and blocked with PBS containing 1% BSA and 1% goat serum for 30 min. The cells on coverslips were incubated with primary antibodies to p65 (1:100, F-6, Santa Cruz) and to pY705-STAT3 (1:200, D3A7, Cell Signaling) for 1 hr, followed by Alexa Fluor 488-conjugated goat anti-mouse F(ab´)2 (1:250, Jackson ImmunoResearch) and Alexa Fluor 647-conjugated goat anti-rabbit F(ab´)2 (1:250, Jackson ImmunoResearch) for 1 h in PBS containing 1% BSA and 0.5% Triton X-100, and then DAPI (300 nM, Molecular Probes) for 5 min. All incubations for coverslips were performed at RT followed by three washes with PBS. Coverslips were mounted with ProLong Gold antifade reagent (Molecular Probes) and placed on slide glass (Sigma). Cells were imaged using a LSM 710 laser-scanning confocal microscope (Carl Zeiss).

### Statistical analysis

All data were analyzed using GraphPad Prism v.4.00 (GraphPad Software Inc, San Diego, CA, USA). Results are presented as means ± SD. Student’s t-test (unpaired, two tailed) and analysis of variance (ANOVA, single factor) were employed for binary and multiple comparisons respectively. The statistical significance was defined as *P* < 0.05.

## Additional Information

**How to cite this article**: Kim, H. S. *et al.* STAT1 deficiency redirects IFN signalling toward suppression of TLR response through a feedback activation of STAT3. *Sci. Rep.* **5**, 13414; doi: 10.1038/srep13414 (2015).

## Supplementary Material

Supplementary Information
