# Supplementary material for: STAT1 deficiency redirects IFN signalling toward suppression of TLR response through a feedback activation of STAT3
Source: Sci Rep. 2015 Aug 24;5:13414. doi: 10.1038/srep13414 (PMC4547106; doi:10.1038/srep13414)
Supplement: Supplementary Information [file srep13414-s1.doc]

**Supplementary information**

**STAT1 deficiency redirects IFN signalling toward suppression of TLR response through feedback activation of STAT3**

Hun Sik Kim, Dong Chan Kim, Hong-Mi Kim, Hyung-Joon Kwon, Soon Jae Kwon, Suk-Jo Kang, Sun Chang Kim & Go-Eun Choi


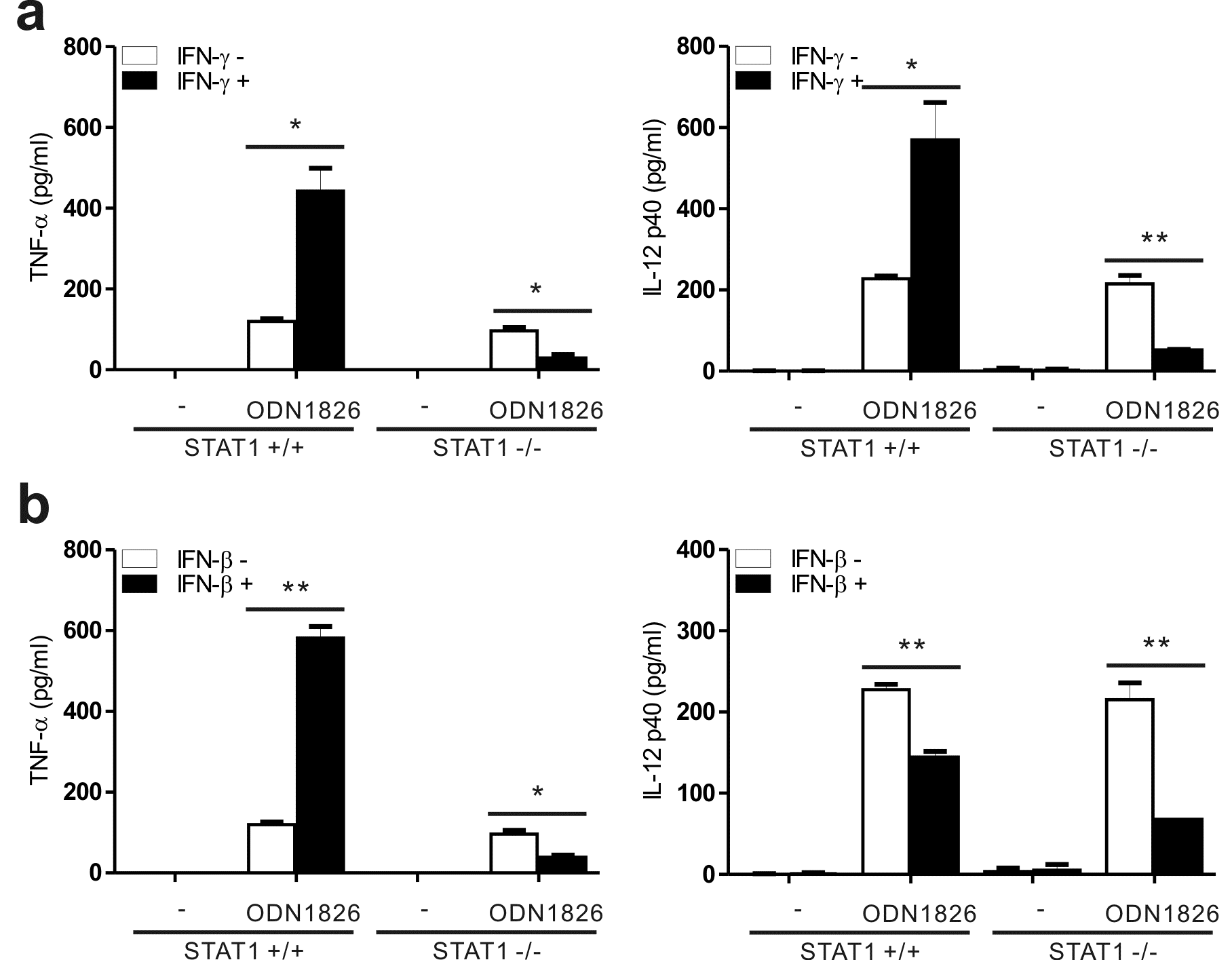


**Figure S1.** IFNs suppress TLR9-induced production of TNF-α and IL-12 by STAT1 deficiency.

Peritoneal macrophages were left untreated (-) or were treated with CpG ODN1826 (2.5 μM) for 8 hr with 10 ng/ml of IFN-γ (**a**) or with 10 U/ml of IFN-β (**b**). The production of TNF-α (*left panel*) and IL-12 p40 (*right panel*) in the supernatant was determined by ELISA. Data represent means±SD of three independent experiments. **P*< .05 and ***P*< .01.


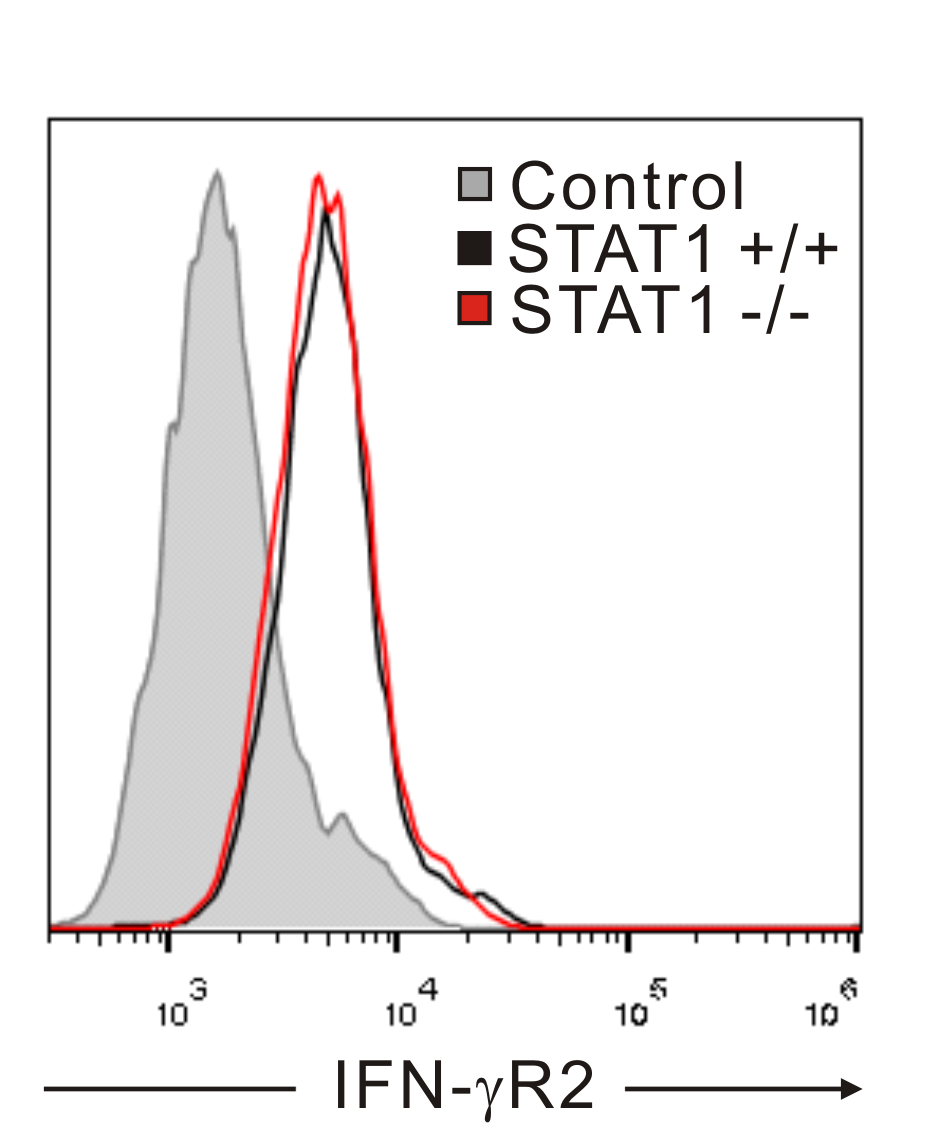


**Figure S2.** The expression level of IFN-γR2 was comparable between wild-type and STAT1-null macrophages.

The surface expression of IFN-γR2 on the macrophages was analyzed using monoclonal antibodies specific for IFN-γR2 or control IgG1.


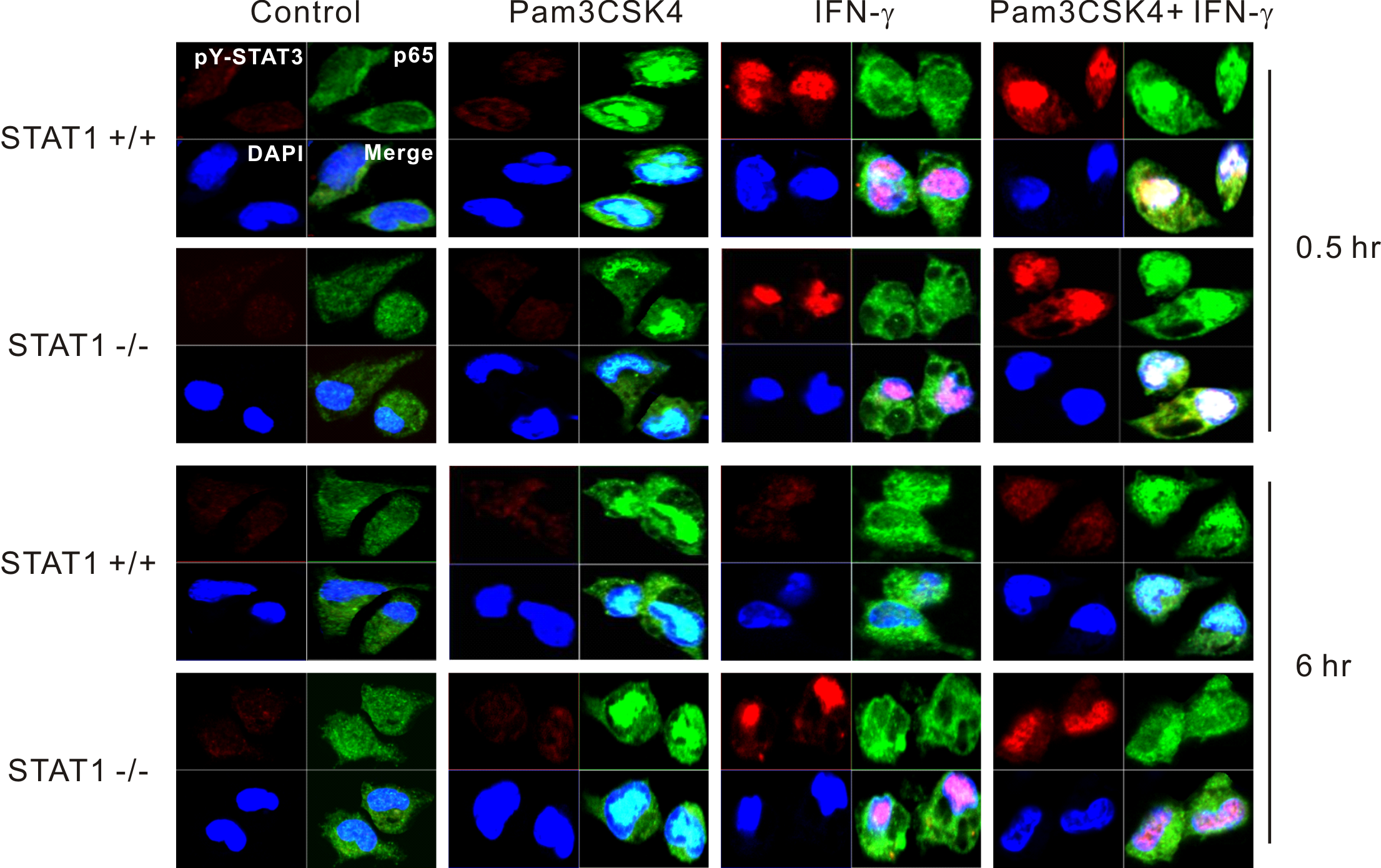


**Figure S3.** Sustained nuclear localization of phospho-STAT3 correlated with the decrease of p65.

Macrophages were left untreated (control) or were treated with Pam3CSK4 (100 ng/ml) for the indicated times with or without IFN-γ (10 ng/ml). Cells were stained using monoclonal antibodies specific for phospho-STAT3 (red), p65 (green), and DAPI (blue), and were then observed under a confocal microscope.


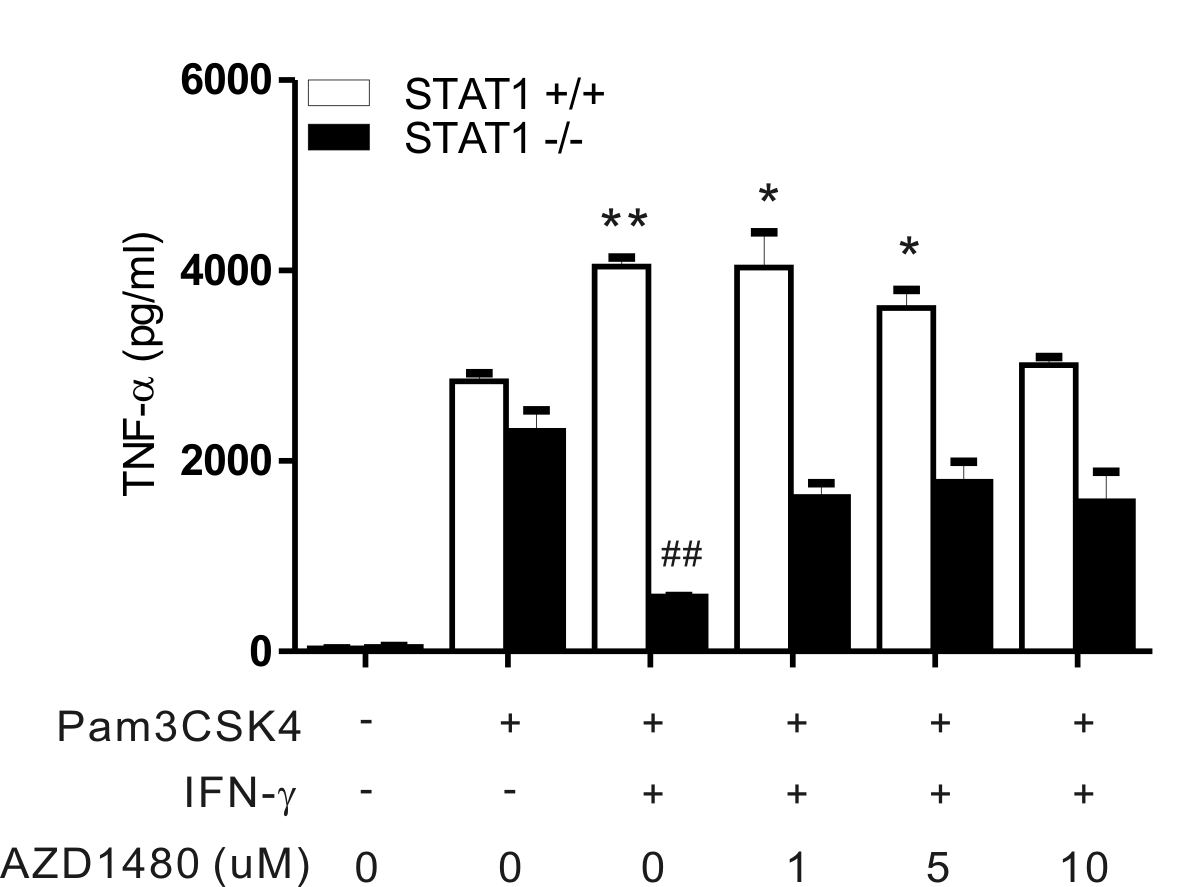


**Figure S4.** Inhibition of JAK2/STAT3 pathway reverses defective TNF-α production and NF-κB signaling.

Macrophages were pretreated with increasing doses of a JAK2/STAT3 pathway inhibitor, AZD1480, for 0.5 hr and were then stimulated with Pam3CSK4 (100 ng/ml) for 8 hr with or without IFN-γ (10 ng/ml). The production of TNF-α in the supernatant was determined by ELISA. Data represent the means±SD of three independent experiments. **P*< .05 and ***P*< .01 for wild-type macrophages. ^##^*P*< .01 for STAT1-null macrophages.


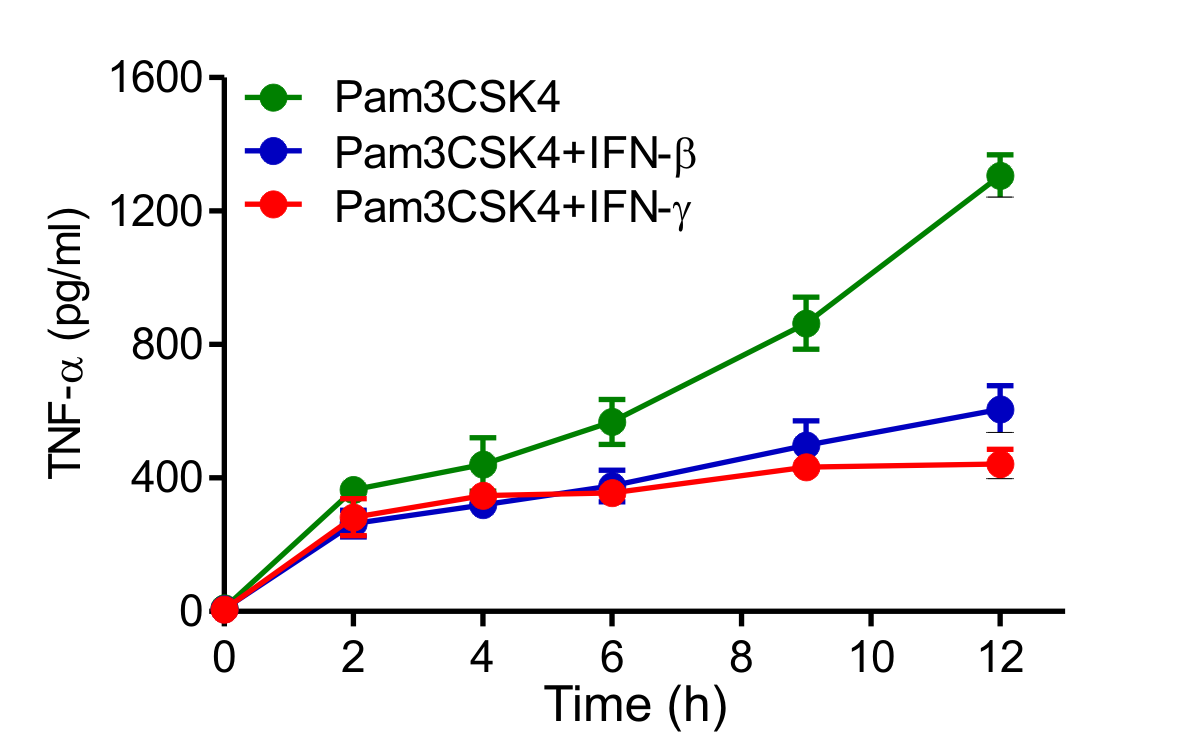


**Figure S5.** STAT1-independent suppression of TLR2 response by IFNs becomes apparent at late time points.

STAT1-null macrophages were incubated with Pam3CSK4 (100 ng/ml) for the indicated times with 10 ng/ml IFN-γ or 10 U/ml IFN-β. The secretion of TNF-α in the supernatant was then determined by ELISA. Data represent the means±SD of three independent experiments.


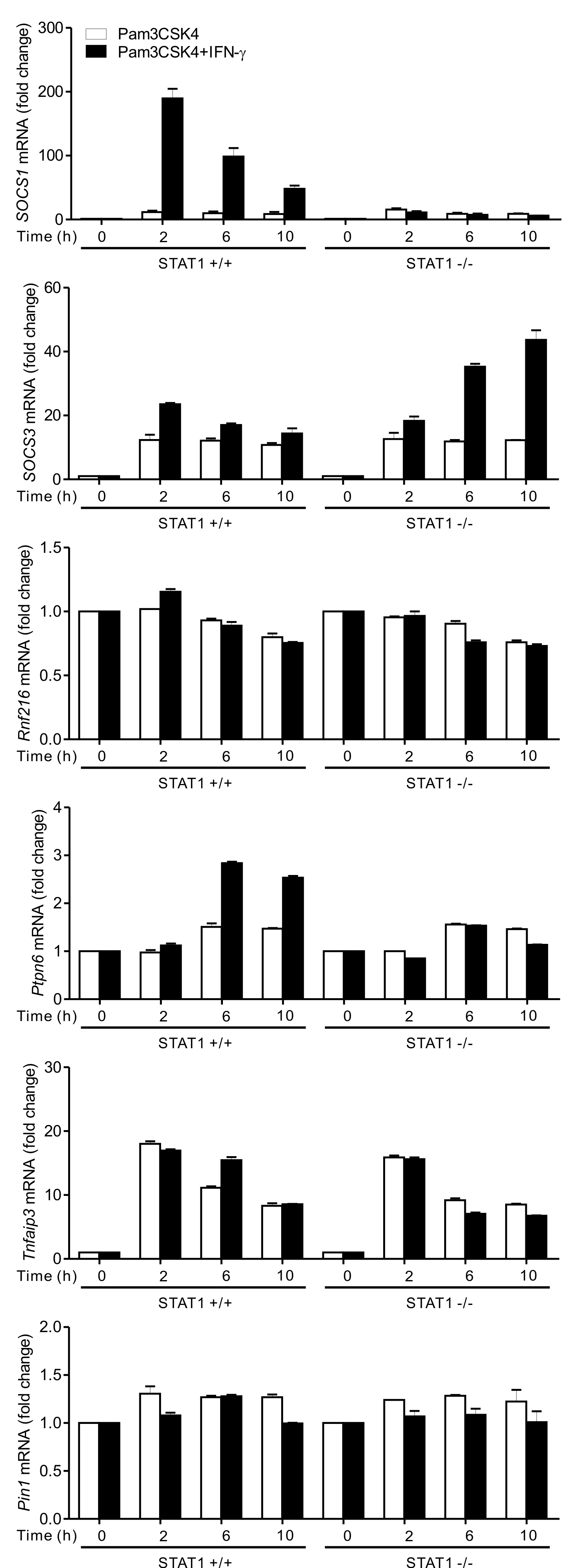


**Figure S6.** The expression levels of several negative regulators for TLR signaling.

Macrophages were incubated with Pam3CSK4 (100 ng/ml) for the indicated times with or without IFN-γ (10 ng/ml). The relative mRNA levels of SOCS1, SOCS3, Triad3a (*Rnf216*), SHP-1 (*Ptpn6*), A20 (*Tnfaip3*), and Pin1 were determined by real-time PCR. Data are representative of three independent experiments.


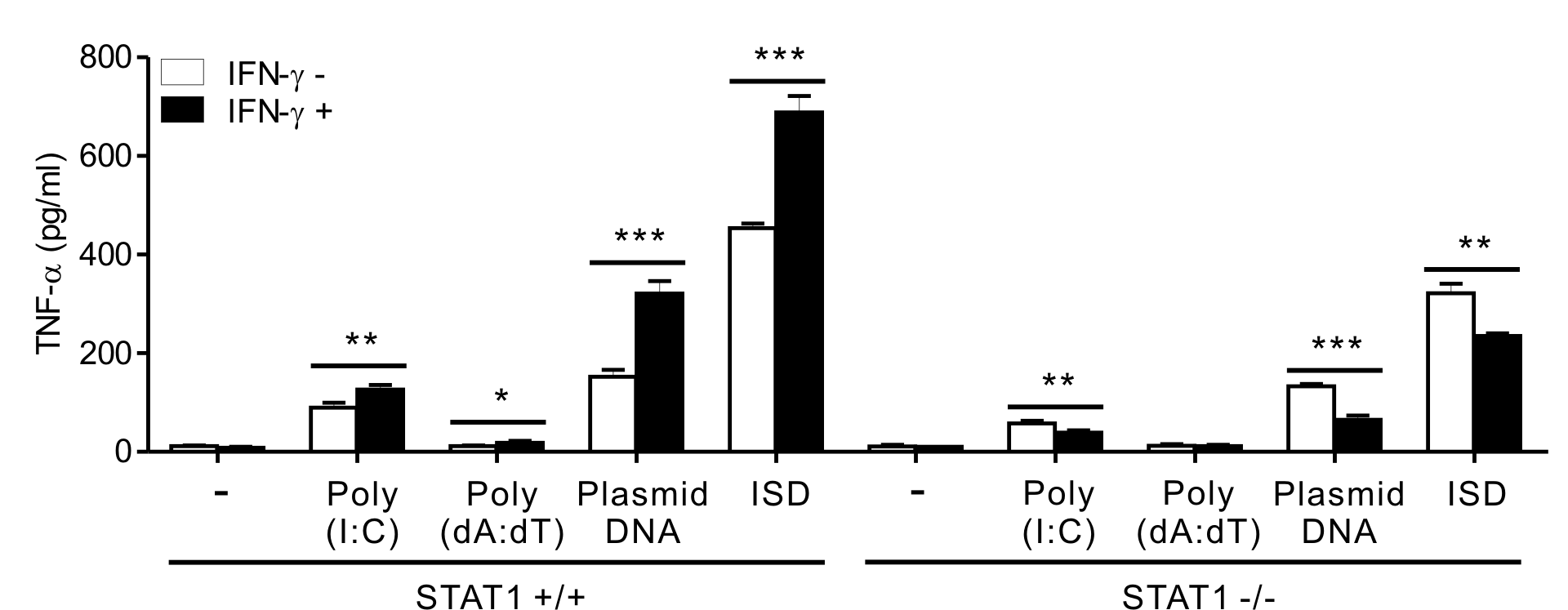


**Figure S7.** STAT1-independent regulation of TNF-α production by IFN-γ in macrophages stimulated with diverse non-TLR agonists.

Macrophages were transfected with different forms of nucleic acids (all at10 μg/ml; poly(I:C), poly(dA:dT), plasmid DNA or ISD) for 24 h with or without 10 ng/ml IFN-γ. The secretion of TNF-α in the supernatant was then determined by ELISA. Data represent the means±SD of three independent experiments. **P*< .05, ***P*< .01 and ****P*< .001.

**Figure S8.** Full-length immunoblots.

Followings are the full-length immunoblots for Figure 5a-5f, Figure 6b, and Figure 7e, 7f.


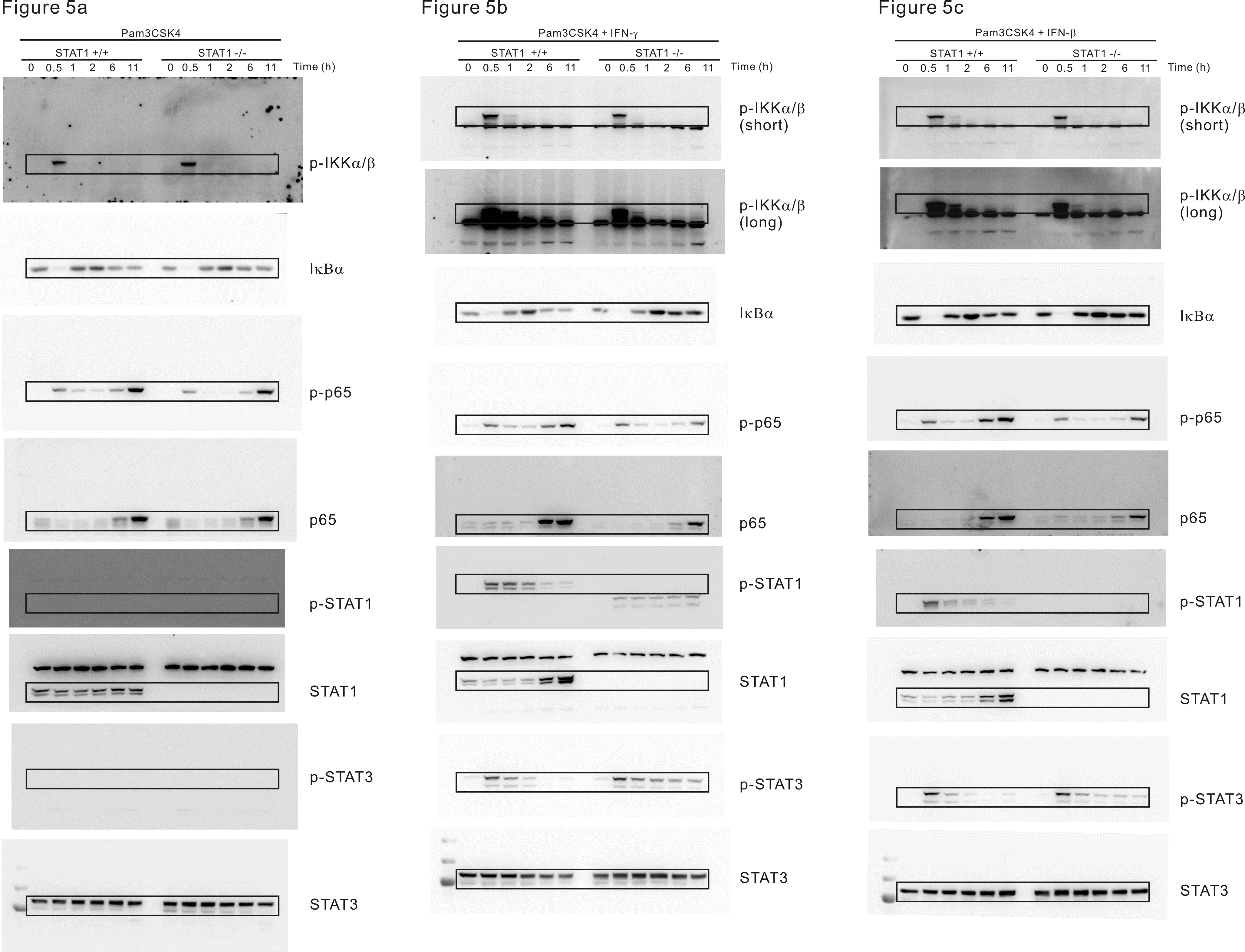


**
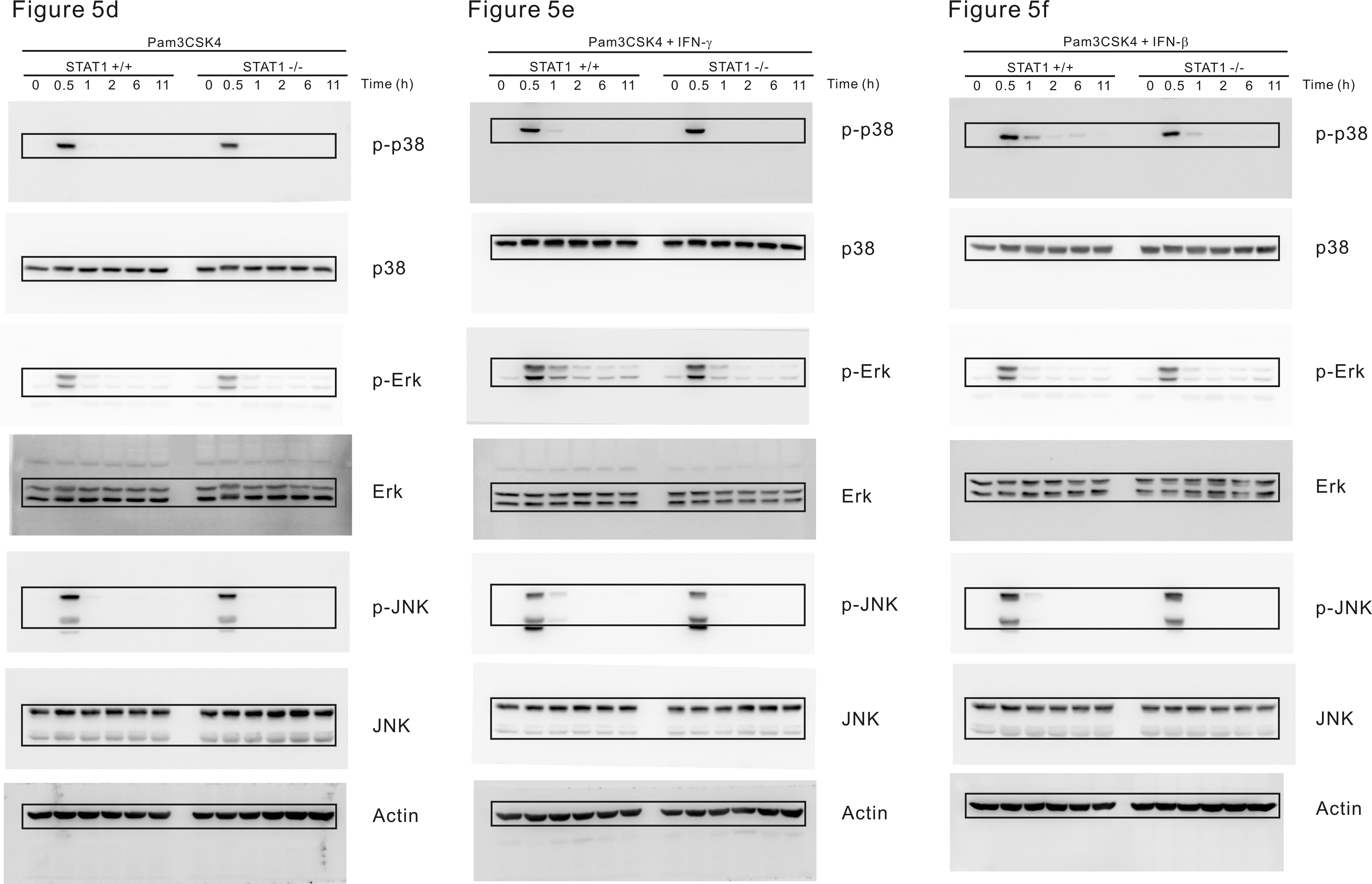
**

**
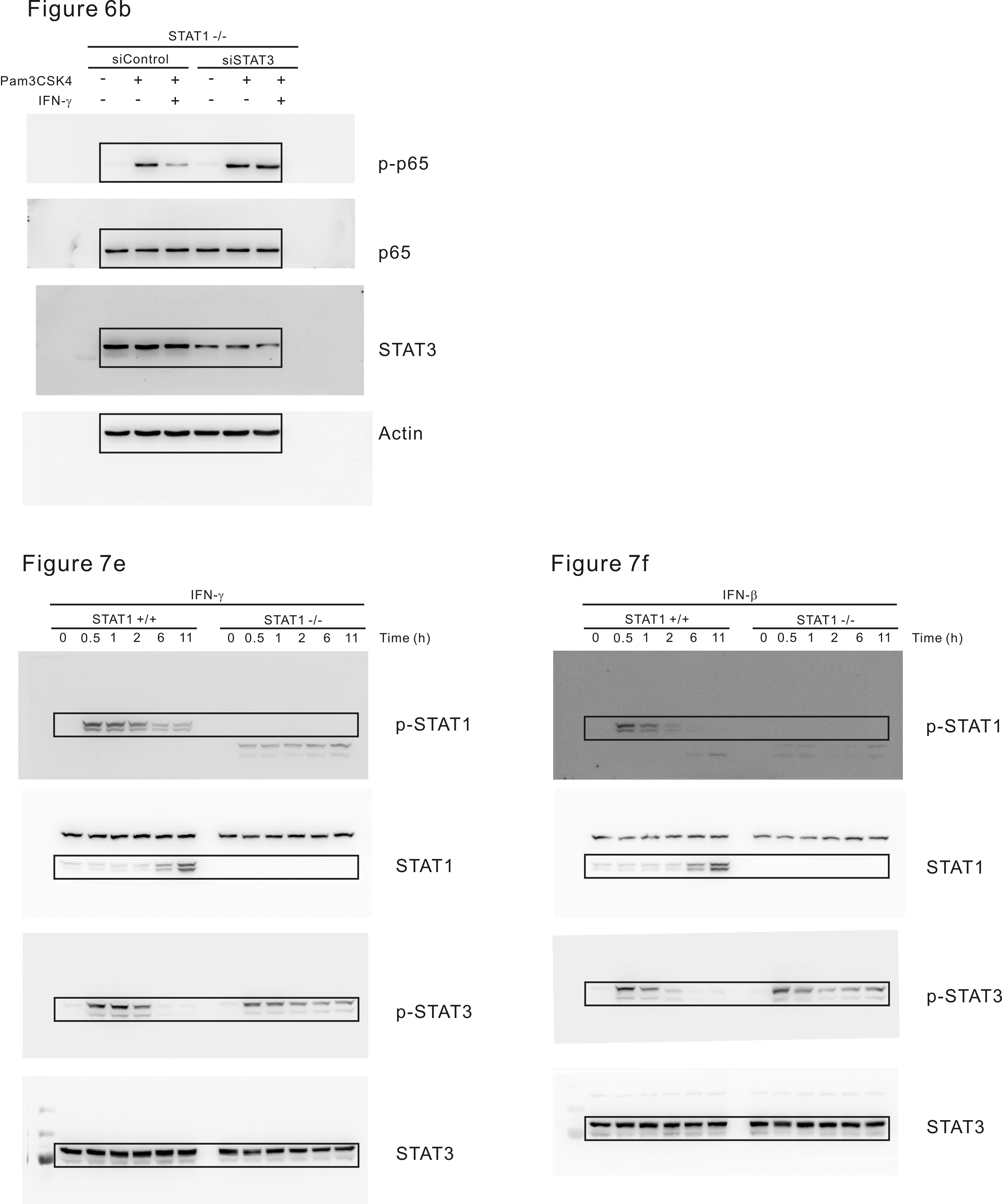
**
